# Description of industrial pollution in Spain

**DOI:** 10.1186/1471-2458-7-40

**Published:** 2007-03-21

**Authors:** Javier García-Pérez, Elena Boldo, Rebeca Ramis, Marina Pollán, Beatriz Pérez-Gómez, Nuria Aragonés, Gonzalo López-Abente

**Affiliations:** 1 Cancer and Environmental Epidemiology Area, National Centre for Epidemiology. Carlos III Institute of Health, C/Sinesio Delgado, 6, 28029 Madrid, Spain

## Abstract

**Background:**

Toxic substances released into the environment (to both air and water) by many types of industries might be related with the occurrence of some malignant tumours and other diseases. The publication of the EPER (European Pollutant Emission Register) Spanish data allows to investigate the presence of geographical mortality patterns related to industrial pollution. The aim of this paper is to describe industrial air and water pollution in Spain in 2001, broken down by activity group and specific pollutant, and to plot maps depicting emissions of carcinogenic substances.

**Methods:**

All information on industrial pollution discharge in 2001 was drawn from EPER-Spain public records provided by the European Commission server. We described the distribution of the number of industries and amounts discharged for each pollutant, as well as emission by pollutant group and the industrial activities associated with each pollutant. Maps of Spain were drawn up, with UTM coordinates being used to plot pollutant foci, and circles with an area proportional to the emission to depict pollution emission values.

**Results:**

The EPER-Spain contained information on 1,437 industrial installations. The industrial plants that discharge pollutant substances into air and water above the pollutant-specific EPER threshold were mainly situated in the Autonomous Regions of Aragon, Andalusia and Catalonia and in Catalonia, the Basque Country and Andalusia respectively. Pollution released in 2001 into air approached 158 million Mt. Emissions into water were over 8 million Mt.

**Conclusion:**

A few single industrial plants are responsible for the highest percentage of emissions, thus rendering monitoring of their possible health impact on the surrounding population that much simpler. Among European countries Spain is the leading polluter in almost one third of all EPER-registered pollutant substances released into the air and ranks among the top three leading polluters in two-thirds of all such substances. Information obtained through publication of EPER data means that the possible consequences of reported pollutant foci on the health of neighbouring populations can now be studied.

## Background

Toxic substances, which are released constantly into the environment (to both air and water) by many types of industrial activities, include a long list of products and pollutants that until now have never been quantified in Spain. Evidence as to the health risk posed by residing in the vicinity of such polluting industries is limited, with cancer and congenital malformations being the most widely studied health problems in the international literature [1-6]. The geographic patterns displayed by certain tumour sites in "small-area" mortality studies in Spain [7-9] suggest that there are environmental factors, closely associated with the territory, which may play an important role in tumour aetiology. It has recently been suggested that exposures deriving from the process of industrial and economic development might have some influence on the appearance of haematological tumours in Spain [10].

In January 2000, the European Council gave a favourable opinion on the implementation of a European Pollutant Emission Register (EPER) [11]. Under the terms of this project, all Member States are required to report to the Commission on emissions to air, soil and water from all agricultural or industrial facilities engaging in one or more activities listed in Annex I to Council Directive 96/61/EC. Industrial activities classified in the EPER fall into the following 6 categories: 1) Energy industries; 2) Production and processing of metals; 3) Mineral industry; 4) Chemical industry and chemical installations; 5) Waste management; and 6) Other activities (which include paper and board production, manufacture of fibres or textiles, tanning of hides and skins, slaughterhouses, intensive poultry or pig rearing, installations using organic solvents, and the production of carbon or graphite). The Directive envisages the reporting of 50 pollutant emissions in excess of a given threshold. The information available allows for different types of industrial activities to be identified and, in addition, contains abundant data on emissions of the pollutant substances to air, water or soil, including the amount released annually.

In February 2004, EPER data on Spain (for 2001) were published. This register traces its origin to the Integrated Pollution Prevention and Control (IPPC) Act 16/2002 of 1 July, governing the authorization of activities falling into 11 categories. The EPER includes all IPPC industrial plants that have exceeded the reporting thresholds for one or more of the pollutants included in EU Decision 2000/479/CE. The aim of this paper was to describe industrial air and water pollution in Spain, broken down by activity group and specific pollutant, and to plot maps depicting emissions of carcinogenic substances. Lastly, the situation in Spain as regards emission of pollutant substances to air is compared to that of other European countries. With the aid of EPER information, the relationship between industrial pollution and public health consequences in Europe can thus be studied by analyzing the influence of spatial distribution of emissions on geographic morbidity and mortality patterns.

## Methods

All information on industrial pollution discharged in 2001 was drawn from EPER-Spain records. This information is public, and the records are accessible as a relational database, from the European Commission server [12]. In every case where the geographic WGS84-projection coordinates for the pertinent industrial installation could be imported and incorporated into a Geographic Information System (GIS), these are shown against the entry concerned.

The data were obtained and processed to include all the information necessary for analysis purposes, in three data files, namely, emissions to air, direct to water and indirect to water (via sewage treatment plants). Although there are 4,949 installations affected by the IPPC Act in Spain, the EPER-Spain included only 1,437, which released pollutants that have exceeded the reporting thresholds in 2001. Data were not published for the remaining installations (3,512) because they did not exceed the reporting thresholds for the pollutants, and consequently they are not considered in this study.

Pollutants/Substances were classified into the following groups:

1) Environmental themes: methane, carbon monoxide, carbon dioxide, hydrofluorocarbons, nitrous oxide, ammonia, non-methane volatile organic compounds (NMVOC), nitrogen dioxide, perfluorocarbons, sulphur hexafluoride, sulphur dioxide, nitrogen and phosphorus.

2) Metals and compounds: arsenic, cadmium, chromium, copper, mercury, nickel, lead and zinc.

3) Chlorinated organic substances: dichloroethane-1,2, dichloromethane, chloroalkanes, hexachlorobenzene, hexachlorobutadiene, hexachlorocyclohexane, halogenated organic compounds, dioxins and furans, pentachlorophenol, tetrachloroethylene, tetrachloromethane, trichlorobenzenes, trichloroethane-1,1,1, trichloroethylene and trichloromethane.

4) Other organic compounds: benzene, toluene, ethylbenzene, xylenes, brominated diphenylether, organotin-compounds, polycyclic aromatic hydrocarbons (PAH), phenols and total organic carbon.

5) Other compounds: chlorides, chlorine and inorganic compounds, cyanides, fluorides, fluorine and inorganic compounds, hydrogen cyanide and PM_10_.

Based on this material, we described the distribution of the number of industries and amounts discharged for each pollutant, as well as emission by pollutant group and the industrial activities associated with each pollutant. Maps of Spain were drawn up, with UTM coordinates being used to plot pollutant foci, and circles with an area proportional to the emission to depict pollution emission values. In order to plot the maps, pollutants classified as group-1, -2A and -2B carcinogens by the International Agency for Research on Cancer (IARC) were selected [13]. Lastly, we compared Spain's percentage emissions to those of European Union countries, focusing on air pollutants for which Spain had some type of emission registered.

## Results

At the date of study, the EPER-Spain contained information on 1,437 plants or industrial installations. Registry data showed that a total of 1,250 plants released pollutant substances to air, 133 direct to water and 164 indirect to water, and that some of these plants discharged substances into both air and water.

### Industrial plants shown by the EPER to discharge pollutant substances into air in Spain, and associated industrial activities

Most of the reported industries were situated in the Autonomous Regions of Aragon, 425 (34%), Andalusia, 208 (17%) and Catalonia, 190 (15%). Industrial activities registering the greatest number of plants were: 'Manure management. Installations devoted to the rearing of poultry and pigs' (723 industries); 'Manufacture of plaster, asphalt, concrete, cement, glass, fibres, bricks, tiles or ceramic products (mineral product industry involving fuel combustion)' (136 industries); and 'Enteric fermentation. Installations devoted to the rearing of poultry and pigs' (75 industries).

### Industrial plants shown by the EPER to discharge pollutant substances directly into water in Spain, and associated industrial activities

The industrial plants registered were mainly situated in Catalonia, 26 (20%), the Basque Country, 23 (18%) and Andalusia, 22 (17%). Industrial activities having the highest number of associated plants were: 'Manufacture of paper, pulp and paper products' (25 industries); 'Manufacture of basic organic chemical products' (19 industries); and 'Manufacture of basic inorganic chemical products or fertilizers' (14 industries).

### Industrial plants shown by the EPER to discharge pollutant substances indirectly into water (via sewage treatment plants) in Spain, and associated industrial activities

The industrial plants were mostly situated in Catalonia, 50 (31%), the Basque Country, 31 (19%) and Andalusia, 18 (11%). Industrial activities having the highest number of associated plants were: 'Manufacture of food products and beverages (in slaughterhouses, plants for the production of milk and other animal or vegetable raw materials)' (33 industries); 'Surface treatment of metals and plastics (metal industries and metal ore roasting or sintering installations. Installations for the production of ferrous and non-ferrous metals)' (29 industries); and 'Manufacture of basic organic chemical products' (24 industries).

Table 1 lists the individual air pollutants and the industrial activities associated with these.

**Table 1:** Description of industrial air pollution, by pollutant and associated industrial activity (2001).

**AIR POLLUTANTS**	**MAIN ASSOCIATED ACTIVITY**^1^	**ACTIVITY GROUP**
**GROUP 1: ENVIRONMENTAL THEMES**		

Methane	Landfills (solid waste disposal on land). Installations for the disposal of non-hazardous waste (>50 Mt/day) and landfills (>10 Mt/day)	Waste management

Carbon monoxide; Perfluorocarbons	Primary and secondary metal production (ferrous and non-ferrous) or sinter plants (metal industry and metal ore roasting or sintering installations)	Production and processing of metals

Carbon dioxide; Sulphur dioxide; Nitrogen dioxide	Combustion installations >300 MW	Energy industries

Hydrofluorocarbons	Manufacture of basic organic chemicals	Chemical industry and chemical installations

Nitrous oxide	Manufacture of basic inorganic chemicals and fertilizers	Chemical industry and chemical installations

Ammonia	Manure management. Installations for poultry (>40000), pigs (>2000) or sows (>750)	Other activities

Non-methane volatile organic compounds	Petroleum product processing (mineral oil and gas refineries)	Energy industries
		

**GROUP 2: METALS AND COMPOUNDS**		

Arsenic; Cadmium; Nickel	Combustion installations >300 MW	Energy industries

Chromium; Copper; Lead; Zinc	Characteristic processes in the manufacture of metals (ferrous and non-ferrous) and metal product (metal industry and metal ore roasting or sintering installations)	Production and processing of metals

Mercury	Manufacture of basic inorganic chemicals and fertilizers	Chemical industry and chemical installations
		

**GROUP 3: CHLORINATED ORGANIC SUBSTANCES**		

Dichloroethane-1,2; Tetrachloromethane	Manufacture of basic organic chemicals	Chemical industry and chemical installations

Dichloromethane; Trichloromethane	Manufacture of pharmaceutical products (solvent use)	Chemical industry and chemical installations

Hexachlorobenzene; Tetrachloroethylene	Characteristic processes in the manufacture of metals (ferrous and non-ferrous) and metal product (metal industry and metal ore roasting or sintering installations)	Production and processing of metals

Dioxins and furans	Combustion installations >300 MW	Energy industries

Trichloroethylene	Paint application (solvent use). Installations for surface treatment or products using organic solvents (>200 Mt/year)	Other activities
		

**GROUP 4: OTHER ORGANIC COMPOUNDS**		

Benzene; Polycyclic aromatic hydrocarbons	Petroleum product processing (mineral oil and gas refineries)	Energy industries
		

**GROUP 5: OTHER COMPOUNDS**		

Chlorine and inorganic compounds; Fluorine and inorganic compounds; PM10	Combustion installations >300 MW	Energy industries

Hydrogen cyanide	Characteristic processes in the manufacture of metals (ferrous and non-ferrous) and metal product (metal industry and metal ore roasting or sintering installations)	Production and processing of metals

^1 ^Industrial activity that generates the highest emissions for each pollutant.

Table 2 shows pollution released in 2001, with a breakdown in quantitative terms for each of the three pollution groups (emissions to air, direct to water and indirect to water) and by pollutant group. The results refer to total emissions for Spain, highlighting the Autonomous Regions with the highest emissions for the respective pollutant groups and their percentages relative to the overall figure.

**Table 2:** Description of pollution released by industrial plants in Spain (2001), by pollutant group.

**AIR POLLUTION**				
	TOTAL SPAIN	REGION WITH HIGHEST VALUE		

	Emission (Mt/year)	Region	Emission (Mt/year)	%

Group 1: Environmental themes	157,585,773	Asturias	24,103,456	(15%)
Group 2: Metals and compounds	1,048	Basque Country	496	(47%)
Group 3: Chlorinated organic substances	240	Basque Country	77	(32%)
Group 4: Other organic compounds	218	Andalusia	109	(50%)
Group 5: Other compounds	47,887	Castile & Leon	11,560	(24%)
				

**WATER POLLUTION (DIRECT)**				

	TOTAL SPAIN	REGION WITH HIGHEST VALUE		

	Emission (Mt/year)	Region	Emission (Mt/year)	%

Group 1: Environmental themes	5,790	Andalusia	1,925	(33%)
Group 2: Metals and compounds	167	Cantabria	99	(59%)
Group 3: Chlorinated organic substances	320	Andalusia	101	(31%)
Group 4: Other organic compounds	31,470	Cantabria	11,930	(37%)
Group 5: Other compounds	7,696,422	Basque Country	6,517,962	(85%)
				

**WATER POLLUTION (INDIRECT)**				

	TOTAL SPAIN	REGION WITH HIGHEST VALUE		

	Emission (Mt/year)	Region	Emission (Mt/year)	%

Group 1: Environmental themes	8,458	Andalusia	7,257	(86%)
Group 2: Metals and compounds	38	Basque Country	20	(53%)
Group 3: Chlorinated organic substances	87	Basque Country	72	(82%)
Group 4: Other organic compounds	53,348	Andalusia	37,558	(70%)
Group 5: Other compounds	366,784	Andalusia	312,204	(85%)

Table 3 gives a detailed description of industrial air pollution in Spain in 2001, as reflected by the EPER, with a breakdown by specific pollutant. While the rows show information relating to the pollutants, the columns reflect the statutory reporting thresholds for the respective pollutant emissions, information for Spain as a whole (total emissions, number of plants that release substances, and mean emission per plant) and information on the Autonomous Regions that registered the highest emission values for each of the pollutants (total emissions, number of plants, and mean emission per plant). The thresholds in the first column of pollutants provide a crude idea of the relative toxicity or importance of the substance. Tables 4 and 5 display the same information as Table 3, but with reference to direct and indirect emissions to water respectively.

**Table 3:** Description of pollution discharged into the air by industrial plants in Spain (2001), by specific pollutant.

**POLLUTANTS**		**TOTAL SPAIN**			**AUTONOMOUS REGIONS WITH HIGHEST EMISSIONS**			
SUBSTANCES	THRESHOLD (Mt/year)^1^	EMISSION (Mt/year)^2^	PLANTS^3^	MEAN EMISSION PER PLANT (Mt/year)^4^	REGION	EMISSION (Mt/year)^2^	PLANTS^3^	MEAN EMISSION PER PLANT (Mt/year)^4^

**GROUP 1: ENVIRONMENTAL THEMES**								

Methane	100	78670	58	1356	Aragon	19048	6	3175
Carbon monoxide	500	243834	56	4354	Asturias	119754	5	23951
Carbon dioxide	100000	155211000	153	1014451	Asturias	23823000	13	1832538
Hydrofluorocarbons	0.1	276	5	55	Catalonia	193	4	48
Nitrous oxide	10	6208	37	168	Castile-La Mancha	1494	6	249
Ammonia	10	24049	815	30	Aragon	10015	399	25
Non-methane volatile organic compounds	100	63781	60	1063	Andalusia	16810	7	2401
Nitrogen dioxide	100	801301	217	3693	Catalonia	394548	33	11956
Perfluorocarbons	0.1	30	4	8	Galicia	18	2	9
Sulphur dioxide	150	1156625	156	7414	Galicia	412414	12	34368
								

**GROUP 2: METALS AND COMPOUNDS**								

Arsenic	0.02	6	33	0.17	Andalusia	2	8	0.20
Cadmium	0.01	5	46	0.11	Basque Country	1	13	0.10
Chromium	0.10	80	40	2	Basque Country	44	11	4
Copper	0.10	29	28	1	Andalusia	15	6	2
Mercury	0.01	3	34	0.08	Catalonia	0.5	8	0.06
Nickel	0.05	171	96	2	Andalusia	42	18	2
Lead	0.20	100	50	2	Basque Country	53	14	4
Zinc	0.20	655	43	15	Basque Country	368	15	25
								

**GROUP 3: CHLORINATED ORGANIC SUBSTANCES**								

Dichloroethane-1,2	1	3	1	3	Catalonia	3	1	3
Dichloromethane	1	61	4	15	Madrid Region	29	1	29
Hexachlorobenzene	0.01	0.03	2	0.02	Extremadura	0.02	1	0.02
Dioxins and furans	0.000001	0.0002	11	0.00002	Basque Country	0.0001	2	0.00005
Tetrachloroethylene	2	72	1	72	Basque Country	72	1	72
Tetrachloromethane	0.10	0.50	1	0.50	Catalonia	0.50	1	0.50
Trichloroethylene	2	81	3	27	Castile & Leon	62	1	62
Trichloromethane	0.50	23	2	12	Andalusia	18	1	18
								

**GROUP 4: OTHER ORGANIC COMPOUNDS**								

Benzene	1	197	20	10	Andalusia	97	5	19
Polyciclic aromatic hydrocarbons	0.05	21	16	1	Andalusia	12	3	4
								

**GROUP 5: OTHER COMPOUNDS**								

Chlorine and inorganic compounds	10	2217	44	50	Andalusia	1582	13	122
Fluorine and inorganic compounds	5	2091	35	60	Andalusia	1122	15	75
HCN	0.2	4	6	0.6	Basque Country	4	6	0.6
PM10	50	43575	119	366	Castile & Leon	11547	10	1155

^1 ^Established air emission threshold or limit value (in Mt/year) for each pollutant. Should any plant exceed this threshold, it must report the release of the pollutant (voluntarily)

^2 ^Amount of pollutant (in Mt/year) released by industrial plants

^3 ^Number of industrial plants that discharge said pollutant

^4 ^Mean emission = Emission/Plants

**Table 4:** Description of pollution directly discharged to water by industrial plants in Spain (2001), by specific pollutant.

**POLLUTANTS**		**TOTAL SPAIN**			**AUTONOMOUS REGIONS WITH HIGHEST EMISSIONS**			
SUBSTANCES	THRESHOLD (Mt/year)^1^	EMISSION (Mt/year)^2^	PLANTS^3^	MEAN EMISSION PER PLANT (Mt/year)^4^	REGION	EMISSION (Mt/year)^2^	PLANTS^3^	MEAN EMISSION PER PLANT (Mt/year)^4^

**GROUP 1: ENVIRONMENTAL THEMES**								

Nitrogen	50	5255	18	292	Andalusia	1759	4	440
Phosphorus	5	535	25	21	Aragon	175	3	58
								

**GROUP 2: METALS AND COMPOUNDS**								

Arsenic	0.005	0.412	11	0.037	Catalonia	0.161	3	0.054
Cadmium	0.005	2	16	0.106	Andalusia	1	8	0.133
Chromium	0.050	7	24	0.293	Catalonia	2	7	0.336
Copper	0.050	9	28	0.309	Basque Country	3	5	0.690
Mercury	0.001	0.281	17	0.017	Cantabria	0.102	2	0.051
Nickel	0.020	9	33	0.275	Aragon	3	2	2
Lead	0.020	3	18	0.187	Andalusia	1	5	0.208
Zinc	0.100	137	37	4	Cantabria	97	6	16
								

**GROUP 3: CHLORINATED ORGANIC SUBSTANCES**								

Dichloroethane-1,2	0.010	0.332	2	0.166	Catalonia	0.308	1	0.308
Chloroalkanes	0.001	0.186	2	0.093	Cantabria	0.161	1	0.161
Hexachlorobutadiene	0.001	0.001	1	0.001	Catalonia	0.001	1	0.001
Halogenated organic compounds	1	319	16	20	Andalusia	100	3	33
								

**GROUP 4: OTHER ORGANIC COMPOUNDS**								

Benzene, toluene, ethylbenzene, xylenes	0.200	64	4	16	Cantabria	47	1	47
Polycyclic aromatic hydrocarbons	0.005	7	6	1	Cantabria	5	2	2
Phenols	0.020	24	17	1	Andalusia	17	4	4
Total organic carbon	50	31375	55	570	Cantabria	11878	6	1980
								

**GROUP 5: OTHER COMPOUNDS**								

Chlorides	2000	7696120	11	699647	Basque Country	6517950	3	2172650
Cyanides	0.050	153	5	31	Asturias	151	1	151
Fluorides	2	149	15	10	Asturias	60	2	30

**Table 5:** Description of pollution indirectly discharged to water by industrial plants in Spain (2001), by specific pollutant.

**POLLUTANTS**		**TOTAL SPAIN**			**AUTONOMOUS REGIONS WITH HIGHEST EMISSIONS**			
SUBSTANCES	THRESHOLD (Mt/year)^1^	EMISSION (Mt/year)^2^	PLANTS^3^	MEAN EMISSION PER PLANT (Mt/year)^4^	REGION	EMISSION (Mt/year)^2^	PLANTS^3^	MEAN EMISSION PER PLANT (Mt/year)^4^

**GROUP 1: ENVIRONMENTAL THEMES**								

Nitrogen	50	6580	11	598	Andalusia	5673	3	1891
Phosphorus	5	1877	26	72	Andalusia	1584	7	226
								

**GROUP 2: METALS AND COMPOUNDS**								

Arsenic	0.005	0.162	4	0.041	Galicia	0.130	1	0.130
Cadmium	0.005	0.370	15	0.025	Cantabria	0.150	1	0.150
Chromium	0.050	5	19	0.261	Basque Country	3	5	0.579
Copper	0.050	4	18	0.221	Basque Country	2	6	0.251
Mercury	0.001	0.078	5	0.016	Basque Country	0.047	2	0.023
Nickel	0.020	8	39	0.206	Aragon	3	2	2
Lead	0.020	2	15	0.141	Cantabria	0.882	1	0.882
Zinc	0.100	18	25	0.738	Basque Country	14	8	2
								

**GROUP 3: CHLORINATED ORGANIC SUBSTANCES**								

Dichloroethane-1,2	0.010	0.023	1	0.023	Catalonia	0.023	1	0.023
Dichloromethane	0.010	0.212	1	0.212	Catalonia	0.212	1	0.212
Chloroalkanes	0.001	0.016	2	0.008	Catalonia	0.016	2	0.008
Halogenated organic compounds	1	87	7	12	Basque Country	72	3	24
								

**GROUP 4: OTHER ORGANIC COMPOUNDS**								

Benzene, toluene, ethylbenzene, xylenes	0.200	10	3	3	Catalonia	9	2	5
Organotin-compounds	0.050	0.154	1	0.154	Andalusia	0.154	1	0.154
Polycyclic aromatic hydrocarbons	0.005	2	7	0.265	Catalonia	2	3	0.502
Phenols	0.020	4	21	0.184	Basque Country	2	3	0.505
Total organic carbon	50	53332	65	820	Andalusia	37557	11	3414
								

**GROUP 5: OTHER COMPOUNDS**								

Chlorides	2000	366750	8	45844	Andalusia	312200	2	156100
Cyanides	0.050	4	4	1	Catalonia	4	2	2
Fluorides	2	30	4	7	Basque Country	26	3	9

Insofar as the pollutant groups were concerned, Table 2 shows that air received the most group-1 pollutants (environmental themes), with the high emissions of carbon dioxide, sulphur dioxide, nitrogen dioxide and carbon monoxide being especially noteworthy in terms of mass (Table 3). In contrast to air, water – both directly and indirectly – received more group-5 pollutants (Other compounds) (Table 2), chlorides in particular (Tables 4 and 5).

**Figure 1:**
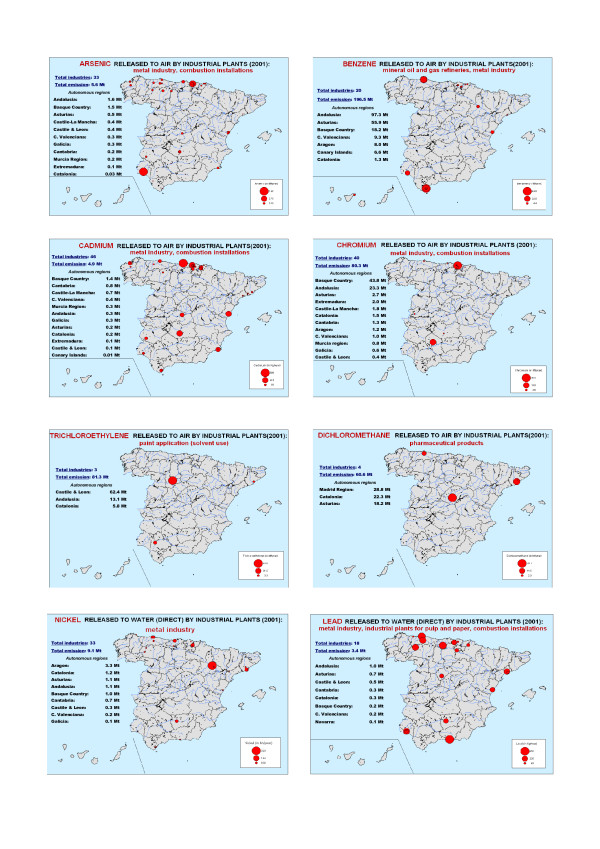
Geographic distribution of industrial foci, by specific pollutant.

Figure 1 shows the geographic distribution of the foci or industrial plants for some carcinogenic pollutants. In the case of air pollutants, the IARC has classified arsenic, benzene, cadmium and chromium as carcinogens (group 1), trichloroethylene as a probable carcinogen (group 2A), and dichloromethane as a possible carcinogen (group 2B). In the case of pollutants discharged direct to water, lead and nickel, both of which have been classified as possible carcinogens by the IARC, are shown [13].

**Figure 2:**
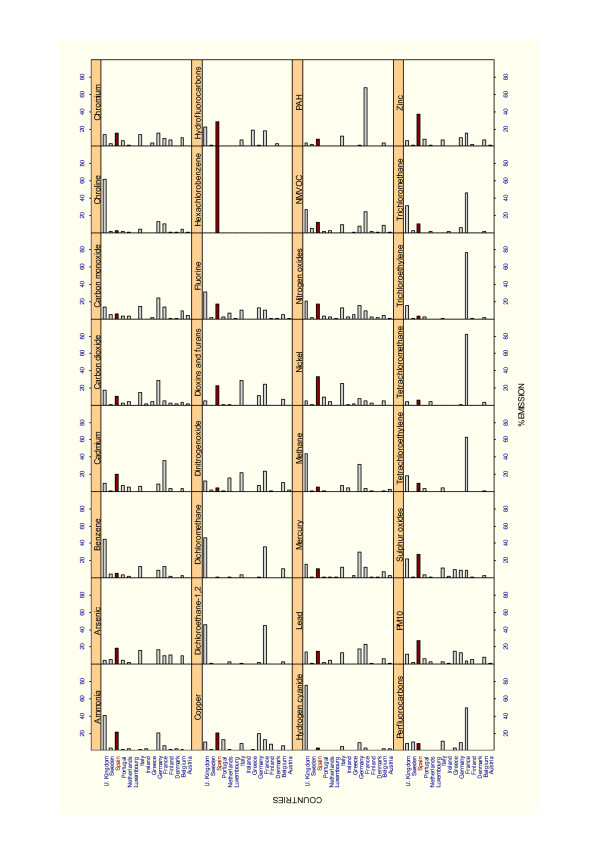
Percentage emissions of pollutant substances released to air in the European Union.

Finally, Figure 2 sets out the percentage emissions recorded by the EPER-Europe of pollutant substances released into the air in the European Union (for 2001). With respect to Spain, note should be taken of the percentage emissions of hexachlorobenzene substances (Spain accounting for 100% of reported emissions), and chromium, nickel and zinc (Spain accounting for around 40% of reported European emissions).

## Discussion

EPER provides data on emissions of key pollutants to air and water from major European industrial facilities. The first set of emissions data, published in February 2004, should be used with caution because of several limitations. One of them is the non-registered industrial plants and/or unquantified emissions, due to the fact that industries are still in the phase of adaptation to EPER regulations and the emission reporting will not become compulsory until 2007. Therefore, data were gathered in 2001 from industries participating in EPER in a voluntary way. In view of the EPER's novelty, the completeness or data quality of this register is not well-known, though given the IPPC's regulatory stance with respect to industrial activity, sufficient quality for the proposed description can be reasonably assumed. However, the 'picture' derived from our study could change in part if completeness and accuracy data were improved.

On the other hand, it should be noted that reported emission data can be obtained by monitoring or modelling. There are three possible codes to indicate the emission determination method for the reported emission data:

Code M: is used when the emissions of a facility are derived from direct monitoring results for specific processes at the facility, based on actual measurements of pollutant concentrations for a given discharge route.

Code C: emission data are based on calculations using nationally or internationally agreed estimation methods (like as fuel used, production rate) and emission factors, which are representative for the industrial sectors. Furthermore, this code is used when the emission calculation methods is obtained from published references [14,15].

Code E: emission data are based on non-standardised estimations derived from best assumptions or expert judgement.

In this first approach, analysis of the EPER 2001 pollution data reveals that industrial air pollution is more intense for substances grouped under: "Environmental themes" in Andalusia, Aragon, Asturias, Castile la Mancha, Catalonia and Galicia; "Metals and compounds" in Andalusia, Catalonia and the Basque Country; "Chlorinated organic substances" in Andalusia, Castile & Leon, Catalonia, Madrid Autonomous Region, Extremadura and the Basque Country; "Other organic compounds" in Andalusia; and "Other compounds" in Andalusia, Castile & Leon and the Basque Country.

On the other hand, industrial pollution discharged directly into water proved more intense for substances grouped under: "Environmental themes" in Andalusia and Aragon; "Metals and compounds" in Andalusia, Aragon, Cantabria, Catalonia and the Basque Country; "Chlorinated organic substances" in Andalusia, Cantabria and Catalonia; "Other organic compounds" in Andalusia and Cantabria; and "Other compounds" in Asturias and the Basque Country.

Lastly, industrial pollution discharged indirectly into water (via sewage treatment plants) was more intense for substances grouped under: "Environmental themes" in Andalusia; "Metals and compounds" in Aragon, Cantabria, Galicia and the Basque Country; "Chlorinated organic substances" in Catalonia and Basque Country; "Other organic compounds" in Andalusia, Catalonia and the Basque Country; and "Other compounds" in Andalusia, Catalonia and the Basque Country.

In Spain, a total of 655 towns have at least one EPER registered facility (excluding farms) in their administrative limit. A total of 215 towns have at least one pollutant facility located within two kilometres from their town centroid (centre point of town) with a population estimated at 500,000 inhabitants. These figures have been calculated after a thorough quality control of the facility UTM coordinates provided by EPER.

As it can be observed from the EPER, a few single industrial plants are responsible for the highest percentage of emissions (Tables 3, 4 and 5). Identification of a small number of high emission plants should elicit implementation of adequately designed health studies in their surrounding.

Analysis of the comparative percentage emissions of pollutant substances released to air in the 15 European Union countries shows that Spain features as a polluter in 32 substances. In percentage terms it ranks: as the leading polluter in 10 of these (arsenic, copper, chromium, nitrogen dioxide, hexachlorobenzene, hydrofluorocarbons, nickel, sulphur oxides, PM_10 _and zinc); as the second-leading polluter in 3 more (ammonia, cadmium and fluorine); and as the third-leading polluter and in a further 7 (non-methane volatile organic compounds, dioxins and furans, polycyclic aromatic hydrofluorocarbons, lead, tetrachloroethylene, trichloroethylene and trichloromethane). According to data released by EPER in 2004, Spain would be the leading polluter in almost one third of all EPER-registered pollutant substances released into the air and ranks among the top three leading polluters in two-thirds of all such substances. It should be noted that this situation can reflect differences in reporting between countries.

With regard to release of substances to air, in which Spain is pre-eminent vis-à-vis the remaining European countries, hexachlorobenzene & a secondary product formed during the manufacture of other chemical substances – has been classified by the IARC as a possible carcinogen to human beings (group 2B) [13]. Its principal health effects stem from ingesting products highly contaminated with this substance [16]. Zinc, whose principal exposure occurs when eating food, drinking water or breathing air polluted with this compound [17], has not been classified by the IARC in terms of carcinogenicity [13], but can nonetheless cause a number of disorders. The IARC has concluded that some nickel compounds are carcinogenic to humans (group 1) and that metallic nickel is possibly carcinogenic to humans (group 2B) [13]. Its most harmful effects are seen when large amounts of compounds of this substance are inhaled [18]. The IARC has decided that chromium compounds (VI) are carcinogenic to humans (group 1) [13] and may increase the risk of contracting lung cancer. The principal health effects follow on from inhaling high levels of this compound [19]. Some studies conducted in Spain have found evidence of risk posed to the population living near industries that release some of these compounds [20-22].

The EPER contains data on the main pollutant emissions to air and water reported by over 10000 medium- and large-sized industrial installations in 17 European countries. Online information searches can be made via the EPER web page, according to type of industrial plant, industrial activity, area, year and pollutant. It is a user-friendly register, from which, not only tables, but also crude data on pollutant emissions and interactive maps can be obtained. This tool enables useful information to be generated in a public health/environmental pollution context, with similar limitations than other registries as, for instance, Toxic Release Inventory (TRI register) in US [23,24]. It should be noted here that the 2001-based data used for this study were in fact published in February 2004, and that in the last two years there may have been corrections by industries to the emission reports submitted for said year. This gives rise to slight discrepancies between the information reported in this paper and that shown on the EPER-Spain web page.

In relation to public health, there has been growing interest in the development of useful statistical methods for detection of patterns of health events linked with pollution sources in recent years. The information obtained through EPER and PRTR would allow to study the consequences of pollutant foci in population health applying focused clustering methods [25,26]. Raised incidence of the health outcomes in the target population living near to the source or directional preference related to a dominant wind direction may provide evidence of such a link [27].

Some authors reported statistically significant associations between lung cancer risk and residential proximity to smelters, complex industrial areas, and other emission sources. There was some evidence that leukaemia and lymphomas occurred in the neighbourhoods that contained industrial sites [1,4,5] The modelling of distance effect to sources of pollution under isotropic or anisotropic assumptions is complex and there are very limited examples in the literature. To date, most pollution source studies concentrate on incidence or mortality of a single disease [26,27].

The EPER is scheduled to be upgraded and replaced by the European Pollutant Release and Transfer Register (PRTR), which will include more comprehensive information on industrial pollution from 91 substances and 65 industrial activities, as well as information on waste management by industrial installations. It will also compile pollutionreports from a range of sources, such as road traffic, aviation, shipping and agriculture. The reports will be annual (rather than triennial as envisaged under the EPER), with the first becoming available in 2007. As from 2009, the PRTR will be accessible by Internet and will have replaced the EPER.

## Conclusion

Information obtained through publication of EPER data means that the possible consequences of reported pollutant foci on the health of neighbouring populations can now be studied, by analyzing geographic mortality patterns of different tumours with reference to the industrial emission of pollutants labelled as carcinogens. This will, in turn, make it possible to quantify the effect exerted by proximity of one or more industries on cancer- and all-cause mortality observed in the surrounding towns and villages.

## Competing interests

The author(s) declare that they have no competing interests.

## Authors' contributions

JGP and GLA conceived the idea and JGP wrote the manuscript. EB contributed to manuscript writing. EB, RR, MP, BPG, NA and GLA revised the manuscript for important intellectual content. All authors contributed to the final version of the manuscript.

## Pre-publication history

The pre-publication history for this paper can be accessed here:


